# The complete chloroplast genome of *Morinda citrifolia* (noni)

**DOI:** 10.1080/23802359.2019.1703586

**Published:** 2020-01-08

**Authors:** Ying-Feng Niu, Jin Liu

**Affiliations:** Yunnan Institute of Tropical Crops, Xishuangbanna, China

**Keywords:** *Morinda citrifolia* L, chloroplast genome, annotation, Rubiaceae

## Abstract

*Morinda citrifolia* L. (Rubiaceae), commonly called noni, is a medicinal plant that is often used as botanical dietary supplement. This study is the first to report and characterize the complete chloroplast genome of *M. citrifolia*. We found that it contains 153,113 bp with a GC content of 38.05%, consisting of two inverted repeat regions (IRs, 25,588 bp), a large single-copy region (LSC, 83,974 bp), and a small single copy (SSC, 17,963 bp) region. One hundred and twenty-five genes were annotated, including 84 protein-coding genes, 33 transfer RNA (tRNA) genes, and 8 ribosomal RNA (rRNA) genes. Phylogenetic analysis showed that *M. citrifolia* and *Gynochthodes officinalis* were closely related. Overall, this study provided a wealth of information for a follow-up phylogenetic and evolutionary study of the Gentianales.

*Morinda citrifolia* L. (Rubiaceae), commonly called noni (Arunachalam 2018), is a medicinal plant that is also used as botanical dietary supplement (Pawlus and Kinghorn 2007). The genus *Morinda* includes approximately 80 species, mostly of Old World origin, and *M. citrifolia* is distributed the Pacific and also in tropical America (Morton 1992). Because of its suggested effects against cancer (Sharma et al. 2016), dyslipidemia (Mandukhail et al. 2010), inflammation (Mckoy et al. 2002), and immunostimulant properties (Brown 2012), many parts of the *M. citrifolia* tree are utilized in medicines, including the roots, leaves, fruit, and seeds (Dixon et al. 1999; Torres et al. 2017).

In recent years, *M. citrifolia* has garnered increasing attention because of its health-promoting properties, which has prompted an increase of research on its phytochemical constituents and biological activity (Pawlus and Kinghorn 2007). However, few studies have been conducted on the nuclear, mitochondrial, and chloroplast genomes of *M. citrifolia*. The chloroplast genome contains abundant genetic information, thus is a powerful tool for studying the evolutionary relationships of species.

Therefore, we have sequenced and analyzed the chloroplast genome of *M. citrifolia*. Healthy young leaves of rooted *M. citrifolia* plants were collected from Xishuangbanna Tropical Flowers and Plants Garden (N 22°01′6.10″ and E 100°47′18.99″). The genomic DNA was isolated from the leaves using a Dneasy Plant Mini Kit (Qiagen). The DNA was stored in an ultra-low temperature specimen library of Yunnan Institute of Tropical Crops (specimen accession number: YITC-2019-FZ-M-002) after quality control. The DNA sequence data of *M. citrifolia* were obtained by the Roche/454 system (Roche 454 Life Sciences) and assembled using the CLC Genomics Workbench v3.6 (http://www.clcbio.com). The chloroplast genome was annotated by DOGMA (Wyman et al. 2004) and manually corrected. The complete chloroplast genome sequence and annotation results of *M. citrifolia* were submitted to GenBank with the accession number of MN699649.

The complete, circular chloroplast genome of *M. citrifolia* contains 153,113 bp including 30.70% A, 31.24% T, 18.68% G, and 19.38% C, with a GC content of 38.05%. The genome is composed of two inverted repeat regions (IRs, 25,588 bp), a large single-copy region (LSC, 83,974 bp), and a small single copy (SSC, 17,963 bp) region. One hundred and twenty-five genes were annotated, including 84 protein-coding genes, 33 transfer RNA (tRNA) genes, and 8 ribosomal RNA (rRNA) genes. In terms of gene function, these protein-coding genes include photosystem I/II, cytochrome b/f complex, ATP synthase, and NADH dehydrogenase.

A phylogenetic analysis was conducted on the complete chloroplast genome sequences of *M. citrifolia* and 19 other Gentianales species, *Jasminum nudiflorum* (Lamiales) was used as the outgroup (Figure 1). MAFFT (Katoh and Standley 2013) and MEGA7.0 (Kumar et al. 2016) were used for multiple sequence alignment and a maximum likelihood (ML) analysis. The results showed that *M. citrifolia* and *Gynochthodes officinalis* were closely related. This study provides a wealth of information for future phylogenetic and evolutionary studies on the Gentianales.

**Figure 1.**
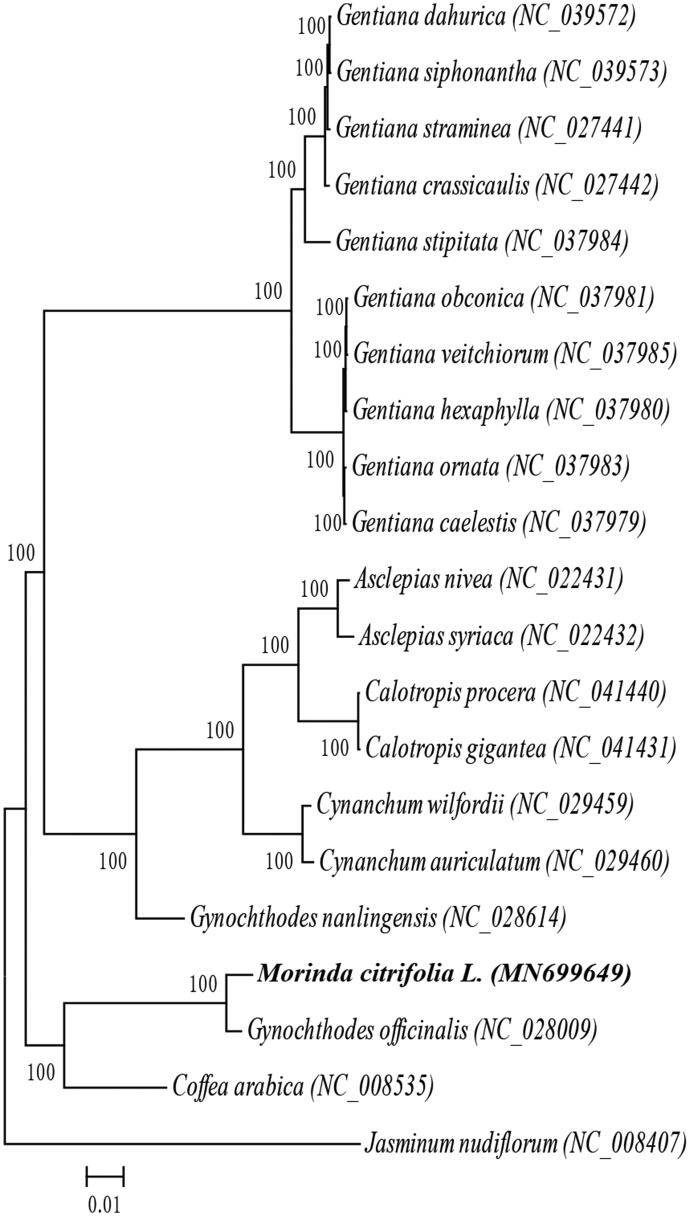
Maximum-likelihood phylogenetic tree of *Morinda citrifolia* L. and 19 other species which belong to Gentianales order based on complete chloroplast sequences, *Jasminum nudiflorum* (Lamiales order) was used as the outgroup. Numbers in the nodes are bootstrap values from 1000 replicates, bootstrap values are shown above the nodes. The species and chloroplast genome accession number for tree construction shown below: *Gentiana dahurica* (NC_039572), *Gentiana siphonantha* (NC_039573), *Gentiana straminea* (NC_027441), *Gentiana crassicaulis* (NC_027442), *Gentiana stipitata* (NC_037984), *Gentiana obconica* (NC_037981), *Gentiana veitchiorum* (NC_037985), *Gentiana hexaphylla* (NC_037980), *Gentiana ornata* (NC_037983), *Gentiana caelestis* (NC_037979), *Asclepias nivea* (NC_022431), *Asclepias syriaca* (NC_022432), *Calotropis procera* (NC_041440), *Calotropis gigantea* (NC_041431), *Cynanchum wilfordii* (NC_029459), *Cynanchum auriculatum* (NC_029460), *Gynochthodes nanlingensis* (NC_028614), *Morinda citrifolia* L. (MN699649), *Gynochthodes officinalis* (NC_028009), *Coffea arabica* (NC_008535), *Jasminum nudiflorum* (NC_008407).
